# Parameterization of disorder predictors for large-scale applications requiring high specificity by using an extended benchmark dataset

**DOI:** 10.1186/1471-2164-11-S1-S15

**Published:** 2010-02-10

**Authors:** Fernanda L Sirota, Hong-Sain Ooi, Tobias Gattermayer, Georg Schneider, Frank Eisenhaber, Sebastian Maurer-Stroh

**Affiliations:** 1 Biomolecular Function Discovery Division, Bioinformatics Institute (BII), Agency for Science Technology and Research (A*STAR), 30 Biopolis Street, #07-01, Matrix, 138671, Singapore; 2 Department of Biological Sciences, National University of Singapore, 14 Science Drive 4, 117543, Singapore; 3 School of Computer Engineering (SCE), Nanyang Technological University (NTU), 50 Nanyang Drive, 637553, Singapore

## Abstract

**Background:**

Algorithms designed to predict protein disorder play an important role in structural and functional genomics, as disordered regions have been reported to participate in important cellular processes. Consequently, several methods with different underlying principles for disorder prediction have been independently developed by various groups. For assessing their usability in automated workflows, we are interested in identifying parameter settings and threshold selections, under which the performance of these predictors becomes directly comparable.

**Results:**

First, we derived a new benchmark set that accounts for different flavours of disorder complemented with a similar amount of order annotation derived for the same protein set. We show that, using the recommended default parameters, the programs tested are producing a wide range of predictions at different levels of specificity and sensitivity. We identify settings, in which the different predictors have the same false positive rate. We assess conditions when sets of predictors can be run together to derive consensus or complementary predictions. This is useful in the framework of proteome-wide applications where high specificity is required such as in our in-house sequence analysis pipeline and the ANNIE webserver.

**Conclusions:**

This work identifies parameter settings and thresholds for a selection of disorder predictors to produce comparable results at a desired level of specificity over a newly derived benchmark dataset that accounts equally for ordered and disordered regions of different lengths.

## Background

### Definition of disorder

Over the last decades, the field of structural biology has gained awareness of the importance of disordered regions or even fully unstructured proteins that participate in biological processes [1-3], culminating in a boom of protein disorder predictor development during the last few years [4]. But even with the growing evidence of the importance of protein disorder in biological events, the precise definition of disorder remains unclear, mainly due to methodological limitations in its detection [5]. Often, disordered segments are called low complexity regions, due to their high propensity for certain amino acid types. Although polar low complexity regions are typically associated with being disordered, the reciprocal is not true. Segments of proteins can be detected as disordered (unstructured), without necessarily having the characteristics of a low complexity region [6,7].

Currently, there is a diverse nomenclature to express similar observations of disorder, such as intrinsically disordered proteins (IDPs), also known as natively disordered, natively unfolded or intrinsically unstructured proteins (IUPs) [5], just to name a few. Whether these terms are used to describe full-length sequences is another issue, as frequently, due to technical limitations, structural evidence is available only for individual domains. Typically, only particular regions of proteins are associated with disorder. Some of these regions may participate in processes where transitions between different conformational states occur, as described in the trinity [8] or quartet models [9].

Consequently, large multi-domain proteins are rarely described structurally as a whole. One well characterized example is the human DNA-repair protein hHR23A [UniProt:P54725], which contains 4 defined structural domains (Ubiquitin-like, UBA1, XPC-binding and UBA2) interconnected through highly flexible (disordered) linker regions [10]. Identification of such flexible linkers is of special importance for eukaryotic proteins that are often built up of multiple domains.

### Disorder vs. low complexity in protein function prediction

The correct identification of protein function in proteomics studies is often a long and tedious effort that requires the usage of several algorithms and predictors on a single sequence in order to converge to a putative function [11]. For example in the ANNIE [12,13] semi-automated pipeline for protein sequence annotation, as a first step, sequences are filtered out for low complexity regions, as they tend to produce a higher number of false positive hits in sequence similarity searches. These compositionally biased regions, often enriched in specific amino acid types, are regularly associated with disorder, and consequently receive less attention, as globular domains are quite well established, easier to characterize and promptly become the centre of attention for function determination.

However, in recent years, disorder has gained the awareness of the protein community as a necessary state for certain groups of proteins to correctly function [4]. In this way, it is not surprising that proteins previously described as denatured are gaining importance among functional proteins, as their disordered nature starts to be associated with biological processes. From the view point of function, disordered regions play a role as mechanical linkers, as flexible segments for entering binding clefts of globular domains, as translocation signals and as regions carrying sites for posttranslational modifications [14,15]. Moreover, several recent papers discuss a wide range of additional functional roles of disordered regions [4,5,16-18].

### An *ideal *benchmark set

Every newly developed predictor is assessed through either cross-validation tests, or direct comparison to other available predictors in benchmarking studies. In either case, having a good and well annotated dataset is a must that is independent of the evaluation means. Misleading annotations can bias the final outcome and, consequently, the judgment of which predictor performs better than another.

To avoid that the evaluation of the predictors could be biased by fully relying on a few available datasets created by the author's predictors, we merged and extended the existing disorder information compiled in the DisProt database [19,20], into one general benchmark dataset, named SL, to include short and long disorder, as well as order information. The SL dataset is, so far, the most complete dataset that accounts for disordered regions of different lengths, as well as regions of missing coordinates annotated as Remark 465 in PDB [21] structures. The addition of order annotation in the SL dataset, based on the availability of structural domains in the PDB, has more than doubled the number of annotated residues from 61837 to 141895. In this way, the SL dataset can be used as a good reference when benchmarking any disorder predictor. For comparison purposes, we also generated a dataset where disorder annotation is based solely on the information of missing coordinates in the PDB annotated as Remark 465. These residues can additionally be annotated as Remark 465 for a few other reasons not limited to their disorder condition, including system-dependent proteolysis, damage of residues through X-ray incidence and incomplete Fourier series. However, these scenarios are expected to be rare and we additionally avoid most of the above biases by requiring a minimum length of 5 for our Remark 465 disorder annotation.

Frequently, varying definitions of disorder are adopted by different groups upon development of a new predictor. DisEMBL [22] is an example of a method that includes three predictors trained to detect three definitions of disorder. Choosing one definition over another constitutes a compromise when analyzing such disordered sequences. Many predictors have been developed as small variations of general methodologies, such as neural networks [22], or sequence profile scoring functions encoding mostly local amino acid composition-based descriptors [23]. In this study, we limited ourselves to evaluating a selection of locally downloadable predictors [22-26] based on distinct methodologies summarized in Table 1. By using the SL dataset, we were able to obtain parameters to compare different methods at the same level of specificity, regardless of an *a priori *disorder definition and evaluate how the methods perform when combined together. These parameter sets are now implemented in our in-house sequence annotation pipeline ANNOTATOR and its public WWW server version ANNIE [11-13,27].

**Table 1:** Summary of the different predictors and their characteristic differences among the methods.

	low sequence complexity	disorder in 3D structures	fully database independent	trained on part of disorder dataset	considers alignment of related sequences
SEG [26,42]	+	-	+	-	-
CAST [23]	+	-	~^#^	-	-
IUPred [25]	-	+	-	-	-
DisEMBL [22]	-	+	-	+	-
DISOPRED2 [24]	-	+	-	+	+^§^

^# ^scores with BLOSUM62

^§ ^through PSI-BLAST

Different concepts of disorder applied during the development of the respective predictors are indicated by markers: "+" indicates that this concept is very relevant for this predictor, "-" indicates that this concept has not been applied and "~" indicates that this concept is indirectly implied.

## Results and discussion

### Comparing the two benchmark datasets

Having a good quality gold standard benchmark dataset is essential when evaluating any predictor. By far, the most complete database of disordered protein segments is provided by DisProt [19,20], the release 4.5 of which was available at the start of this work. At the same time, DisProt should not be directly used as a benchmarking set, since the amount of residues annotated as ordered (1.2%) is by an order of magnitude lower than the number of residues (24.7%) with disorder annotation (missing negative dataset).

Therefore, we generated two new datasets, named Remark 465 and SL, which stands for short and long disorder, as described in the method section. The percentage of residues in each dataset is displayed in Table 2. As a service for the community, both datasets can be downloaded from http://mendel.bii.a-star.edu.sg/SEQUENCES/disorder/ and as electronic supplement of this paper (Additional files 1 and 2). In brief, we tried to match the protein sequences in DisProt r4.5 with sequences of known structures and found 364 entries in DisProt r4.5 that match at least one entry in the Protein Structure Database [21]. Our Remark 465 dataset comprises these 364 sequences where the residues matched by the known protein structures are classified as ordered and the residues covered by Remark 465 annotations in these structures are assigned to the disorder class.

**Table 2:** Percentage of residues in the different datasets.

Dataset	Number of residues with disorder annotation (%)	Number of residues with order annotation (%)	Non-annotated residues (%)	Total number of residues in dataset
DisProt r4.5 (Jul 2008)	24.7	1.2	74.1	239120 (in 520 proteins)
Remark 465	7.2	53.7	39.1	164793 (in 364 proteins)
SL	26.3	33.0	40.7	239120 (in 520 proteins)

The SL dataset comprises the DisProt release 4.5 data in addition to residues in the same proteins annotated as having an ordered 3D structure found by similarity searches among sequences of known tertiary structure. Since some of these structures contain unidentified segments (Remark 465 regions), the number of residues with disorder annotation in SL is slightly larger than in DisProt

As a result, 53.7% of its residues are annotated as ordered against 7.2% as disordered. This number is comparable to the 6% of disordered residues from the 96 targets used in the disorder prediction benchmark of CASP7 [28]. The assessment of disorder prediction has been successfully introduced during the 5^*th *^Critical Assessment of Techniques for Protein Structure Prediction (CASP5) [29] and established since then during the following CASP experiments [28,30]. However, datasets based exclusively on Remark 465 are often restricted to shorter disordered regions, do not easily account for longer ones and do not include information when disorder plays a functional role, as considered in DisProt.

**Figure 1:**
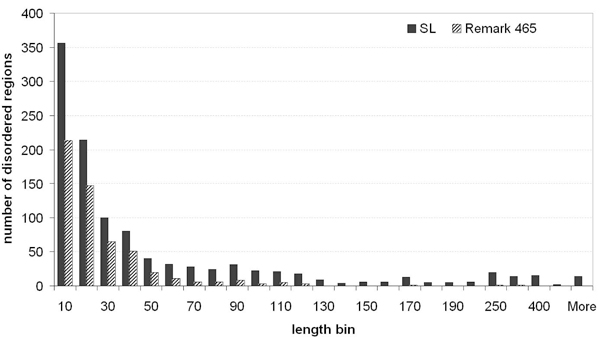
**Length distribution of disordered regions**. The distribution of disordered regions according to their length is shown for each dataset: SL and Remark 465.

The SL dataset is the unification of DisProt r4.5 and our Remark 465 datasets. In conflicting cases of annotations, the disordered description was maintained in SL and the information about order was discarded. We considered it important to maintain the disorder annotation also for regions that fold into structures under specific conditions, such as binding with another globular domain or in a crystal context. The SL dataset contains more short disordered regions than the Remark 465 dataset (see Figure 1 for the distribution of the disordered regions according to their length). This comes from the fact that the SL dataset has additional disorder annotation that is not limited to the missing coordinates in the PDB. Further, it comprises very long disordered regions, or completely disordered proteins, classified as Intrinsically Disordered Proteins (IDPs). One such example is the Bcl2 antagonist of cell death [UniProt:Q61337] that contains the BH3 motif. This protein, of approximately 200 residues, is annotated as having an α-helical region comprising 27 structural residues [PDB:2BZW, chain B] in the Remark 465 dataset with no disorder information, while in the SL dataset, the complete sequence is annotated as disordered, given that the BH3 motif is known to form its helical structure upon interaction with other anti-apoptotic members of the Bcl-2 family [31].

**Figure 2:**
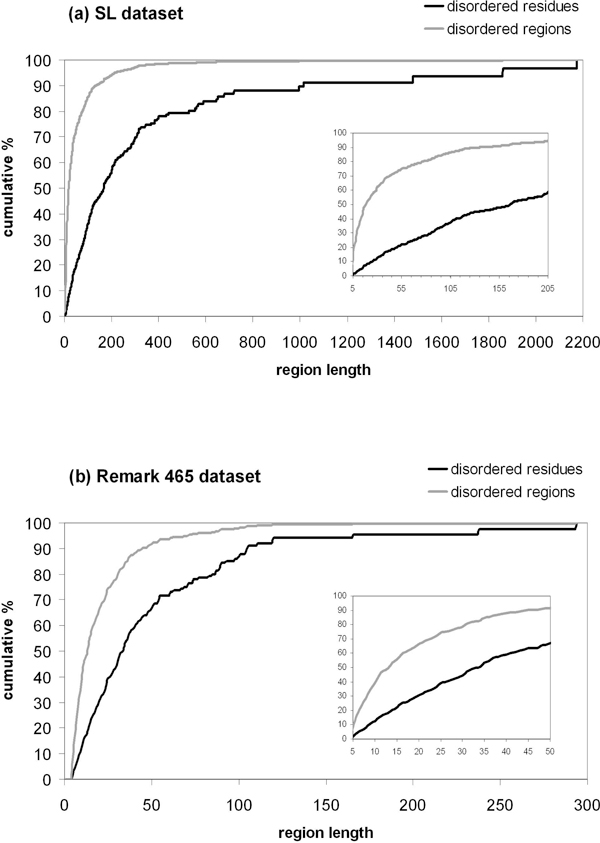
**Cumulative percentage of disorder as a function of region length**. The cumulative distributions of the number of disordered residues and the number of disordered regions for the two differently annotated datasets are shown. (a) SL dataset and (b) Remark 465.

For comparing the length of disordered regions relative to the amount of disordered residues in each of the datasets, we calculated the cumulative percentage distributions shown in Figure 2. We see that, in the SL dataset, 50% of disordered regions are shorter than 19 residues (see Figure 2a). However, this number accounts for only 8% of the total number of disordered residues in the dataset. In fact, 50% of disordered residues are found in regions up to 166 residues in length, which covers 92% of disordered regions. The remaining 50% of disordered residues are found in very few, but even longer stretches of sequences.

Comparatively, in the Remark 465 dataset, 50% of disordered regions are shorter than 13 residues long (see Figure 2b). Therefore, half of the disordered regions in each SL and Remark 465 datasets are comparable to each other relative to the length of short disordered regions. However, in the Remark 465 dataset, these short regions account for 18% of disordered residues compared to 8% in the SL dataset, confirming that the SL has longer disordered regions annotated. If we now consider 50% of disordered residues in Remark 465, we find that they are all in regions of up to 33 residues in length. There were only 3 disordered regions of length 166 and longer in the Remark 465 dataset, against 91 in the SL one. These were the precursor of fibrinogen alpha chain from chicken (P14448), the precursor of human epidermal growth factor receptor (P00533) and the transcription initiation factor IIA large subunit from Baker's yeast (P32773). While the dataset of CASP7 has only 2 regions of length >40 residues, our Remark 465 dataset has 65 regions >40 residues. This was achieved by considering a much larger number of structural domains in comparison to CASP7. The SL dataset has 335 such regions.

**Figure 3:**
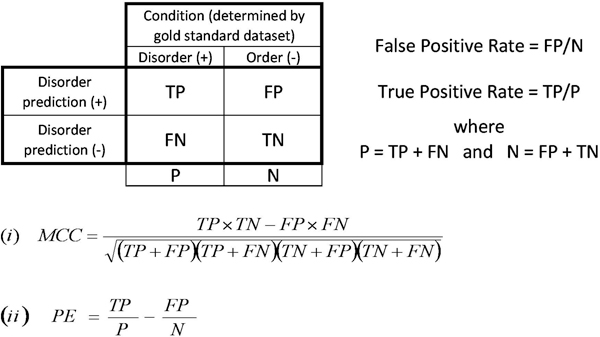
**Contingency table and common performance measurements**. The abbreviations are for the number of true positive (TP), false positive (FP), false negative (FN) and true negative (TN) predictions. The False Positive Rate (FPR) was calculated as FP/(FP+TN) and the True Positive Rate (TPR) as TP/(TP+FN). The Matthews Correlation Coefficient (MCC) is shown in equation (*i*) and the probability excess (PE) in equation (*ii*).

Clearly, if either DisProt r4.5 or Remark 465 is independently considered as a benchmark dataset while evaluating disorder predictors, the results might be affected by the skew in the distribution between order and disorder information (Table 2). In this regard, ROC curves provide a good solution when assessing the predictors, as they are insensitive to changes in the ratio between the numbers of order and disorder examples [32]. However, several other measurements such as accuracy, probability excess (PE) [33] or the Matthews Correlation Coefficient (MCC) [34] (see Figure 3 and Methods section) are altered upon shifts of this ratio [32]. To overcome this issue and to have a more complete dataset where both order and disorder information is considered, we created the SL dataset. Here, we have 26.3% of residues annotated as disorder against 33% in the ordered state (Table 2).

### Evaluating the predictors with the two benchmark datasets

**Figure 4:**
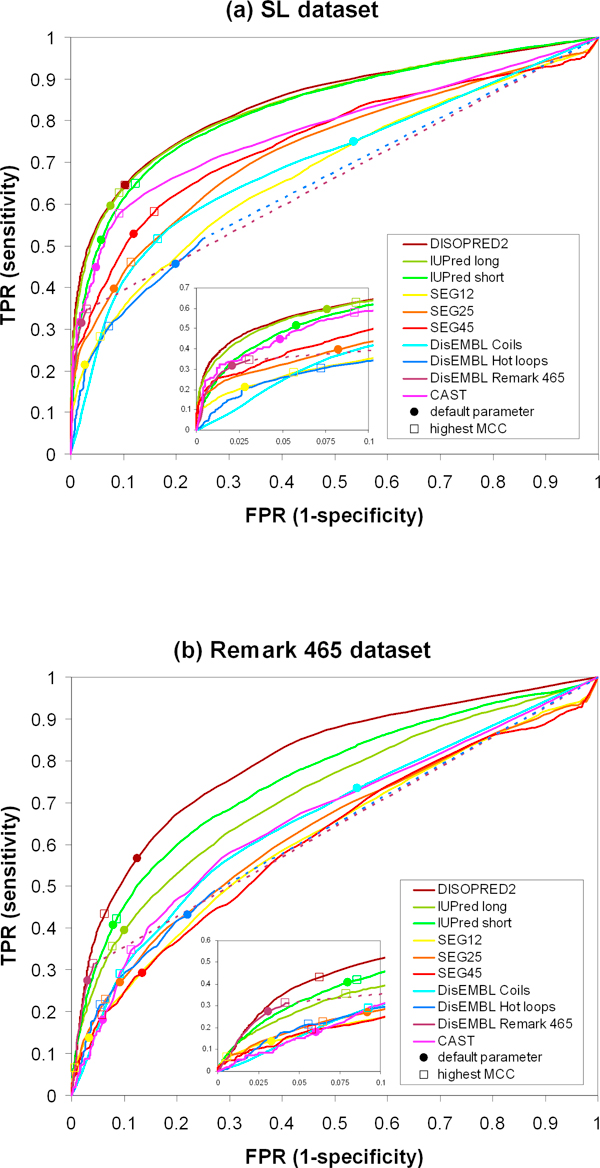
**ROC curves for ten different predictors**. (a) Benchmark against SL dataset. (b) Benchmark against Remark 465 dataset. Filled circles are the points at default threshold and empty squares at highest Matthews Correlation Coefficient. Dotted lines are the straight continuation of the last measurable data point for DisEMBL predictors to point (1,1) in ROC space. FPR and TPR are false positive rate and true positive rate, respectively.

We evaluated the performance of five selected disorder predictors over a wide range of parameters and the results are shown as ROC curves in Figure 4. ROC curves provide a guideline to select a compromise between the amount of false positive predictions (1 - specificity) and the level of sensitivity (correct predictions) achieved by the classifier. If one wants to compare the performance among different predictors, the same specificity level should be taken into consideration. We list the parameters for each disorder predictor where the amount of false positive prediction is the closest to 5% in Tables 3 and 4. Unfortunately, we could not produce a parameter setting for DisEMBL Remark 465 that corresponds to a specificity level of 95%. Instead, our tables show values corresponding to 3-4% false positive predictions for this method.

**Table 3:** Performance benchmark with the SL dataset under parameters at comparable high specificity level (~0.950).

Method	threshold	sensitivity	specificity	MCC	PE
DISOPRED2	0.08	0.557	0.947	0.559	0.504
IUPred long	0.54	0.544	0.948	0.550	0.492
IUPred short	0.51	0.491	0.948	0.507	0.440
CAST	40	0.448	0.951	0.474	0.399
*DisEMBL Rem465*	1	0.348	0.969	0.418	0.317
SEG45	3.30;3.60	0.368	0.950	0.402	0.318
SEG25	2.94;3.24	0.335	0.946	0.364	0.281
SEG12	2.29;2.59	0.268	0.950	0.305	0.218
DisEMBL Hotloops	2.7	0.259	0.949	0.295	0.208
DisEMBL Coils	1.94	0.251	0.948	0.286	0.200

Predictors were run with parameters tuned to achieve a comparable specificity level close to 0.950, which corresponds to ~5% of false positive predictions. For DisEMBL Remark 465 (in italic), parameter tuning only allowed a specificity of 0.969 as closest value to our criterion. Ranking is based on the Matthews Correlation Coefficient (MCC) but remains essentially unchanged for other performance measures such as probability excess (PE).

**Table 4:** Performance benchmark with the Remark 465 dataset under parameters at comparable high specificity level.

Method	threshold	sensitivity	specificity	MCC	PE
DISOPRED2	0.11	0.393	0.950	0.388	0.344
*DisEMBL Rem465*	1	0.316	0.958	0.338	0.274
IUPred short	0.55	0.328	0.947	0.318	0.275
IUPred long	0.59	0.285	0.948	0.277	0.233
DisEMBL Hotloops	3	0.204	0.950	0.196	0.153
SEG25	2.91;3.21	0.197	0.951	0.194	0.149
SEG12	2.29;2.59	0.188	0.943	0.162	0.130
SEG45	3.27;3.62	0.175	0.949	0.162	0.124
DisEMBL Coils	1.96	0.167	0.950	0.155	0.117
CAST	48	0.154	0.949	0.136	0.103

Predictors were run and ranked as described in Table 3 but over the Remark 465 dataset which puts more emphasis on short disorder inside or flanking globular structures. For DisEMBL Remark 465 (in italic), parameter tuning only allowed a specificity of 0.958 as closest value to our criterion.

Obviously, the data shows that the ranking among the methods is essentially independent of the performance measurement used (sensitivity, MCC or PE) both in Tables 3 (dataset SL) and 4 (dataset Remark 465). Thus, the ratio between the numbers of ordered and disordered residues in the datasets has no effect on the relative ranking of methods. This is possible because the specificity level has been kept constant among all methods (see also Additional file 3, Figure 1). To summarize, keeping constant the specificity or sensitivity levels is a precondition for a fair comparison among methods (see Additional file 3, Figure 1).

At approximately 95% specificity (Tables 3 and 4), DISOPRED2 [24] is the method with the highest percentage of correct predictions in both datasets (55.7% and 39.3% in the SL and Remark 465 datasets, respectively) and it is followed very closely by IUPred long [25] in the SL dataset and IUPred short [25] in the Remark 465 dataset. By comparing the two settings of IUPred (long and short), we see that IUPred long performs better than IUPred short in the SL dataset, while the opposite consistently occurs in the Remark 465 dataset (see also Figure 4). As expected by the different nature of the two available detection settings, IUPred short was able to better identify Remark 465 disordered regions than its long segment counterpart. But for detecting disorder in general, including long disordered regions, IUPred long should be the preferred setting. DisEMBL Remark 465 also performs quite well and it is among the top 3 methods for the identification of short disordered regions (see Figure 4b and Table 4). However, this is not surprising, given that DisEMBL Remark 465 was trained to detect this definition of disorder.

Another picture emerges when the default settings are used (see Tables 5 and 6). Because of the different levels of specificity obtained under the default settings of each predictor, the simple ranking according to any measurement is compromised and different rankings are produced by following various performance indicators. For instance, if we were to rank the methods by highest sensitivity, DisEMBL Coils could be placed on the top row of Table 6. This, however, would come at the cost of accepting a very high false positive prediction rate (54.3%). One should note here that DisEMBL Coils is trained to detect loops/coils that can be but are not necessarily required to be disordered. Hence, this predictor alone is considered to be promiscuous [22].

**Table 5:** Performance benchmark with the SL dataset under parameters at default values.

Method	threshold	sensitivity	specificity	MCC	PE
DISOPRED2	0.05	0.645	0.897	0.567	0.541
IUPred long	0.5	0.596	0.924	0.560	0.520
IUPred short	0.5	0.513	0.942	0.515	0.454
CAST	40	0.448	0.951	0.474	0.399
SEG45	3.40;3.75	0.527	0.880	0.441	0.407
DisEMBL Rem465	1.2	0.314	0.979	0.407	0.293
SEG25	3.00;3.30	0.396	0.917	0.374	0.313
SEG12	2.20;2.50	0.213	0.972	0.293	0.184
DisEMBL Hotloops	1.4	0.456	0.801	0.275	0.257
DisEMBL Coils	1.2	0.750	0.464	0.220	0.214

All predictors were run over the SL set using their respective default settings and ranked by the MCC. These settings produce results of varying levels of specificity which makes their ranking more dependent on the used overall performance measure (e.g. MCC or PE).

**Table 6:** Performance benchmark with the Remark 465 dataset under parameters at default values.

Method	threshold	sensitivity	specificity	MCC	PE
DISOPRED2	0.05	0.566	0.874	0.373	0.441
DisEMBL Rem465	1.2	0.273	0.969	0.330	0.242
IUPred short	0.5	0.406	0.920	0.327	0.326
IUPred long	0.5	0.394	0.899	0.276	0.293
SEG25	3.00;3.30	0.269	0.908	0.182	0.177
SEG12	2.20;2.50	0.136	0.967	0.160	0.103
DisEMBL Hotloops	1.4	0.432	0.780	0.159	0.211
CAST	40	0.179	0.940	0.146	0.119
SEG45	3.40;3.75	0.293	0.865	0.141	0.157
DisEMBL Coils	1.2	0.733	0.457	0.124	0.190

Predictors were run and ranked as described in Table 5 but over the Remark 465 dataset (short disorder). As in Table 5, rankings differ if based on MCC or PE.

It is notable that the ranking of methods in accordance with various performance indicators is different for the two datasets SL (Table 5) and Remark 465 (Table 6). This is a result of differing ratios of numbers of ordered and disordered residues in the two datasets (about 1:1 in SL and about 7:1 in Remark 465).

We also explored the parameter settings where the Matthews Correlation Coefficient (MCC) is maximized for each method. As the MCC approaches zero, the predictions are likely to be random, but as its value gets closer to 1, the higher the correlation between the predictions and the annotation in the benchmark dataset. In this case, by selecting the threshold value producing the highest MCC in ROC space, we are not focusing on which parameter to use to compare the different methods with each other at the same specificity level (or error rate), as described previously, but rather which settings to apply if one wants to extract the best general predictor performance as judged by MCC. In this study, the highest MCC ranges from 0.30 to 0.57 and 0.17 to 0.39 when considering the ten different predictors benchmarked with the SL and Remark 465 datasets, respectively (see Tables 7 and 8). These discrepancies in correlation coefficients between the 2 datasets clearly indicate that the results of a benchmark in a biased dataset such as the Remark 465 should only be taken into account in particular scenarios such as the identification of short disordered regions prior to structural elucidation. Because of the different class distributions between the two datasets, the MCC should not be used to compare performance across the benchmark datasets. If the aim is to determine the general disposition of a protein to be disordered, which includes short and long regions, the results of a benchmark with a dataset such as the SL should be considered.

**Table 7:** Performance benchmark with the SL dataset under parameters that produced the highest Matthews Correlation Coefficient (MCC).

Method	threshold	sensitivity	specificity	MCC	PE
DISOPRED2	0.05	0.645	0.897	0.567	0.541
IUPred long	0.48	0.627	0.907	0.564	0.534
IUPred short	0.41	0.649	0.877	0.546	0.526
CAST	24	0.578	0.908	0.522	0.485
SEG45	3.45;3.75	0.582	0.841	0.442	0.423
DisEMBL Rem465	1	0.348	0.969	0.418	0.317
SEG25	3.05;3.35	0.460	0.885	0.387	0.345
DisEMBL Coils	1.8	0.515	0.835	0.373	0.350
SEG12	2.35;2.65	0.282	0.943	0.308	0.225
DisEMBL Hotloops	2.3	0.306	0.928	0.304	0.233

Predictors were run under parameters that produced the highest MCC over the SL dataset to benchmark their maximally possible performance. Rankings by MCC and PE differ only slightly. Interestingly, the identified optimal parameters (in regard to MCC performance over our dataset) often differed from the default parameters of the respective programs, except for DISOPRED2.

**Table 8:** Performance benchmark with the Remark 465 dataset under parameters that produced the highest Matthews Correlation Coefficient (MCC).

Method	threshold	sensitivity	specificity	MCC	PE
DISOPRED2	0.09	0.433	0.937	0.388	0.370
DisEMBL Rem465	1	0.316	0.958	0.338	0.274
IUPred short	0.49	0.421	0.914	0.328	0.335
IUPred long	0.53	0.355	0.921	0.284	0.276
CAST	24	0.349	0.887	0.218	0.235
DisEMBL Coils	1.9	0.290	0.907	0.200	0.198
DisEMBL Hotloops	2.9	0.217	0.944	0.198	0.161
SEG25	2.95;3.25	0.229	0.935	0.191	0.164
SEG12	1.95;2.25	0.065	0.994	0.170	0.059
SEG45	3.30;3.60	0.192	0.942	0.166	0.134

Predictors were run and ranked as described in Table 7 but over the Remark 465 dataset including parameter optimization for this dataset with focus on short disorder.

From the analysis of ROC curves in Figure 4, we see that the predictions occur at different levels of specificity and sensitivity under default parameters (filled circles). These default parameters did not always produce the best possible performance as judged by MCC (empty squares). The only exception is DISOPRED2 if applied over the SL dataset. At the same time, DISOPRED2 exhibited the highest MCC among all other methods (0.567), followed very closely by IUPred long (0.564) (see Table 7). The performance of IUPred long under default parameters was sufficiently close to the one of highest MCC. Basically, DISOPRED2 and IUPred long have comparable performances if both short and long disordered regions are taken into consideration, as in the SL dataset (Figure 4a). Additionally, DISOPRED2 performed better than other methods for short disorder predictions (Figure 4b). Here, only IUPred short had its default performance close to the one judged by the highest MCC (Figure 4b).

The fact that DISOPRED2 and IUPred long have a comparable performance in the SL dataset provides an argument to select IUPred long over DISOPRED2, when speed in calculation is an issue. This helped in the selection of IUPred long over DISOPRED2 in the implementation of algorithms in the ANNIE webserver [12,13], given that DISOPRED2 considers sequentially similar sequences through PSI-BLAST generated alignments, augmenting considerably the amount of computational time (~40 seconds per protein just for the PSI-BLAST step).

Finally, evaluating the predictors with two datasets gives a general overview and provides a desirable framework to obtain settings at comparable levels of specificity. These settings can be finally applied in semi-automated pipelines, such as in our in-house ANNOTATOR/ANNIE platform, to improve sequence function predictions in large-scale studies. The next step is to evaluate how combinations of such methods compare to individual performance, as addressed in the following section.

### Predicting long disorder

In the functional annotation process of uncharacterized protein sequences coming from full genome sequencing projects, the determination of long disordered regions is far more important than the detection of short disordered segments. The assumption is that long disordered regions are disordered because of the absence of a proper hydrophobic core that would force them into a stable globular structure. These long disordered regions rival globular domains in length and it is questionable whether they are suitable for distant homology searches. We tested the predictors for the detection of long disordered regions by modifying our SL dataset to annotate as disorder only the regions of length 40 and above. This subset is called LD40. As we modify the annotation in the positive set only, the settings by which we obtain the desired specificity level of ~95% is the same as in Table 3. The ranking is displayed in Table 9. We find that the IUPred long method obtained the highest ranking in this task followed by DISOPRED2. All other methods performed clearly worse. As expected, SEG, which is commonly used for low complexity filtering in sequence similarity searches, ranks better with longer averaging window. The three DisEMBL variants are not useful for the detection of long disorder regions.

**Table 9:** Performance benchmark with the LD40 dataset under parameters at comparable high specificity level.

Method	threshold	sensitivity	specificity	MCC	PE
IUPred long	0.54	0.597	0.948	0.602	0.545
DISOPRED2	0.08	0.587	0.947	0.591	0.534
IUPred short	0.51	0.521	0.948	0.539	0.469
CAST	40	0.510	0.951	0.535	0.462
SEG45	3.30;3.60	0.419	0.950	0.454	0.369
*DisEMBL Rem465*	1	0.359	0.969	0.437	0.328
SEG25	2.94;3.24	0.373	0.946	0.406	0.319
SEG12	2.29;2.59	0.291	0.950	0.333	0.240
DisEMBL Coils	1.94	0.263	0.948	0.302	0.211
DisEMBL Hotloops	2.7	0.262	0.949	0.301	0.210

Predictors were run and ranked as in Tables 3 and 4 with parameters tuned to produce comparable high specificity of ~0.950 (~5% of false positive predictions) but over the LD40 dataset which only includes long disordered regions (length of 40 residues and above). For DisEMBL Remark 465 (in italic), parameter tuning only allowed a specificity of 0.969 as closest value to our criterion.

While this manuscript was in preparation, a new predictor specialized in long disorder regions, named IUPforest-L, became available [35]. As this predictor could only be accessed through a webserver, we were forced to limit ourselves to obtain only a few data points displayed in the ROC graph of Figure 2 in Additional file 3. Although the results ultimately appear to be the best ones for the detection of long disorder, this outcome should be considered carefully. When looking at individual proteins, small globular domains tend to be predicted as disordered under the default settings of IUPforest-L. Examples of such wrong predictions include multi-domain proteins such as the human DNA-repair protein hHR23A and protein G.

### Low complexity and disorder: combining pairs of methods

One might think that the combination of disordered predictors leads to improved performance since they are based on different definitions of disordered regions. Such an approach has already been suggested in the literature [36-38]. For example, SEG [26] is a very common and widely used method to filter out low complexity regions in sequence homology searches. This has enormously facilitated the identification of new globular regions in proteins [11]. However, not all disordered regions in proteins are low complexity regions [7]. Therefore, SEG does not perform very well in this study (Figure 4). We see that the bigger the window size parameter in SEG, the better its performance in the SL dataset (Figure 4a, Tables 3 and 5). Can SEG successfully complement, for example, disorder predictors derived from 3D structures or sequence similarity information [6]?

If the predictors identified different regions of disorder due to their different methodological approaches, the combination of two methods together should outperform any individual method. It is not unusual that a more recently developed predictor is claimed to identify new disordered regions that were previously missed by more established methods in the literature such as DISOPRED2 and that, in a combination, they should be beneficial for improved prediction performance [37].

**Figure 5:**
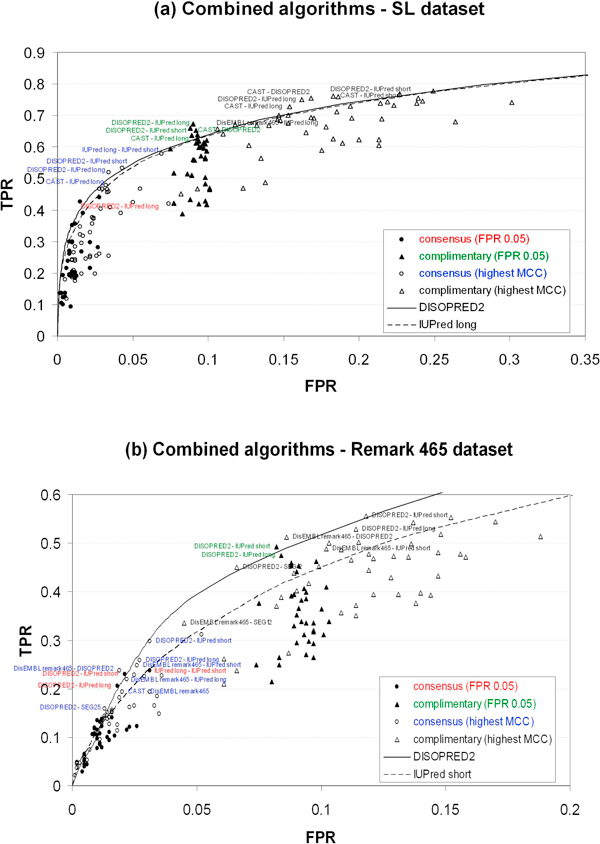
**Performance of combined algorithms**. Consensus and complementary predictions at highest MCC and false positive rate at ~0.05. (a) SL dataset. (b) Remark 465 dataset. ROC curves for DISOPRED2 and IUPred long and short were used as reference. Only the data points closer and above the DISOPRED2 curve are labelled. FPR and TPR are false positive rate and true positive rate, respectively.

In this work, we explored the combined performance of any pair of disorder prediction algorithms. In contrast to previous work [33,36], we used the parameters that reproduce the same level of specificity for each method at a false positive rate of 0.05 (Tables 3 and 4). In addition, we also combined them applying the parameters where the highest MCC was obtained (see Tables 7 and 8). The results of this investigation are summarized in Figure 5. As a trend, the combination of two methods either through consensus or complementary predictions results in a slight improvement of performance compared to single methods. We find that DISOPRED2, which has ranked quite well in the individual comparison to other methods, can only be slightly improved through combination with almost any method but, if at all, the best effect is achieved with IUPred long, CAST [23] or DisEMBL Remark 465. On the other hand, only the combination of IUPred long with either CAST (for the SL dataset) or DisEMBL Remark 465 (for the Remark 465 dataset) reaches the single method performance of DISOPRED2. This is of interest due to the long computation time required for DISOPRED2 compared to other methods.

As expected, the result of combining the methods through consensus predictions is seen as a shift towards less false positive predictions in ROC space, contrary to the complementary one, that is shifted towards more false positive predictions (Figure 5). Still, neither consensus nor complementary predictions resulted in outstanding performances.

### Disorder in complete proteomes: the need of experimental validation to improve future predictions

Undoubtedly, there is a strong necessity of more reliable annotation of disordered regions in protein sequences. So far, the DisProt database is the most complete compilation of disorder annotation in proteins, given the current experimental limitations in disorder detection and its curated nature. Its latest release contains 523 sequences with about 37% of them coming from humans. However, if we now consider the latest estimate of the human proteome in the IPI [39] and calculate the percentage of human sequences that have been annotated as disordered in DisProt, we obtain the extremely low fraction of only ~0.25%. In previous work using DISOPRED2, the approximate fraction of residues predicted as disordered in humans was 21% [24]. We used IUPred long to predict disorder in a non-redundant set of 56915 sequences of the IPI human database and obtained a comparable percentage of disordered residues (23% at an expected rate of 5% false positive predictions) as in the previous work with DISOPRED2 [24] (see Table 10). The list of actual predictions is available from the website that is associated with this paper http://mendel.bii.a-star.edu.sg/SEQUENCES/disorder/.

**Table 10:** Estimated disorder frequencies in the human proteome with IUPred long.

IUPred long threshold	minimum length to consider region as disordered	% of sequences with disorder	% of disordered residues
0.50	0	82.6	26.7
0.50	10	62.0	22.8
0.50	30	38.8	17.5
0.54	0	78.8	23.3
0.54	10	57.3	19.7
0.54	30	34.9	14.9

A subset with 56915 non-redundant sequences (< 98% identity) from the human IPI v3.54 database was used to estimate frequencies of disordered residues and sequences with disorder predicted by IUPred long at different thresholds (0.5 ... default; 0.54 ... estimated 5% false positive rate)

Clearly, identification through experimental validation of all these disorder predictions could increase quite considerably the number of proteins annotated as disordered and the understanding of their role in biological processes, so far mainly found to participate in signalling, recognition and regulation [4,5]. At the same time that predictors are developed to automate the detection and annotation of protein disorder, there is a general saturation and little improvement with newer disorder predictors [28]. Apparently, more experimentally supported disorder annotation appears necessary, despite the big effort in attempting to identify functional classes associated to disorder by using theoretical arguments [40].

### Comparison to other benchmark studies

The largest database of disorder to date, DisProt, should not be taken directly as a benchmark set due to the lack of coverage of the order annotation. On the other hand, previously used benchmark sets are based on disorder annotated as Remark 465 in the PDB, which provides very good quality order annotation. However, such sets typically only cover short disordered regions in close vicinity to or inside otherwise globular structures, which is only one of the many flavours of disorder in proteins [41]. To provide a compromise between the best sources for disorder (DisProt) and order (PDB), we complemented the DisProt annotation with known ordered regions if respective atom coordinates of DisProt proteins were recorded in the PDB.

Furthermore, disordered regions of minimum length 5 annotated in PDB as Remark 465 that map to unannotated regions in DisProt proteins were added as well. This procedure of extending the annotation of DisProt proteins has the additional advantage that both ordered and disordered regions are taken from the same protein set which means that any compositional bias resulting from different taxonomic distributions or subcellular localization sampling (e.g. the amino acid composition might differ slightly between nuclear, cytosolic and extracellular proteins) is avoided in our benchmark set.

There have been many benchmark studies on disorder predictors over the last ten years, with CASP being a major reference in the field, despite its limitation due to a dataset built up in its majority by crystallographic structures that are generally known to display mainly short disordered regions. Typically, benchmark studies are accompanied by the development of a new predictor and are aimed at showing its performance in contrast to previously existing methods.

In a recent review [36], a few practical examples were used to show how the combination of different methods improves disorder prediction, as the methods are generally biased towards detecting different definitions of disorder. Here, we saw that the combination of methods did not result in outstanding performances, which would be expected if they were detecting different flavours of disorder upon a benchmark with a general dataset such as our SL one. Despite their biases in disorder detection, they were not complementary to each other to the extent that it would be worth considering a pair of methods since they just marginally increase the number of correct predictions.

Most benchmarks also use many different scores to rank the predictors due to the natural difficulty in defining what constitutes a good performance in ROC space. We have already underlined previously that certain performance measures such as MCC depend on the composition of the database, *i.e.*, the ratio of residues with disordered and ordered annotation. In contrast, the probability excess is an example that is independent of this ratio [33]. In the latter study, the authors rank and compare the performance of the different methods regardless of a fixed specificity setting. This ultimately generates misinterpretations of what is judged as best, as only data points at default parameters are considered, while the methods should be compared at the same specificity level. As we have seen previously, the influence of choice of the performance measure to rank the methods becomes almost irrelevant, when they are compared at the same specificity level.

## Conclusions

The lack of a precise definition of disorder is a major problem that directly affects the dataset used for benchmarking. Consequently, it strongly influences any measurable outcome, such as accuracy, MCC, probability excess, including the specificity level of the predictor. In this work, we have derived a general dataset based on all currently available data that includes most or all flavours and lengths of disorder for a thorough evaluation of disorder predictors. We complemented the curated disorder annotation in the DisProt database with order annotation from well defined structures in PDB, as well as associated short disorder regions. Thereby, we essentially doubled the number of annotated residues compared to the original DisProt 4.5 annotation.

Ideally, predictors should not be ranked using a single performance measurement at their default settings, since these typically produce results in different areas of the ROC space. Instead, we suggest to test and identify settings where the specificity or sensitivity over the same unbiased dataset is directly comparable. In our in-house sequence analysis pipeline ANNOTATOR/ANNIE [11-13,27] and proteome-wide studies, predictions at high specificity are required and we present threshold and parameter settings for the tested predictors in this scenario.

In our work, we showed that combining different methods yields a positive improvement but the results are not dramatically different, especially if one wants to use the methods for the identification of disorder in complete proteomes. So far, DISOPRED2 has been the method that best unifies all information, but it is limited because of the time demanding PSI-BLAST step for proteome-wide studies. Interestingly, the faster IUPred long that uses a totally different approach was essentially performing similarly well and, at the same time, it is computationally cheaper.

Although the number of annotated residues in the SL dataset appears substantial, it has to be assumed that still only a small fraction of actually disordered regions are currently covered by these annotations. Besides the large number of unannotated disordered regions reliably predicted at thresholds with high specificity, one cannot exclude the possibility that additional flavours of disorder exist that are not properly captured by any of the existing experimental methods.

## Methods

### Generating the benchmark datasets

We generated two datasets for benchmarking the disorder predictors, where each residue was assigned as ordered, disordered or unknown, calling them Remark 465 and short and long (SL) disorder datasets.

The Remark 465 dataset encloses 364 protein sequences, which represent sequences from the DisProt release 4.5 database [19,20], where we could identify a structural domain in the PDB [21]. The regions in the amino acid sequence where the atomic coordinates were solved experimentally and available in the PDB were classified as ordered, while parts of the sequence where the atomic coordinates were not solved, likely due to its disorder condition, with minimum length 5 and annotated in the PDB as Remark 465, were assigned as disordered. This dataset is comparable, in its disorder definition, to the 96 targets used in CASP7 to assess protein disorder prediction [28].

The short and long disorder (SL) dataset includes all 520 protein sequences from the DisProt release 4.5 database [19,20]. Here, the DisProt disorder annotation prevails over structural (order) information. In addition, stretches of sequences with at least five consecutive residues annotated under Remark 465 in the PDB [21] were also assigned as disordered in the SL dataset.

The binary class distribution in terms of percentage of residues classified as ordered or disordered can be seen in Table 2.

### Selection of predictors for benchmarking

There are currently over 20 different disorder predictors available, based on variations of general methodologies, such as machine learning approaches including neural networks, support vector machines, etc. using feature descriptors ranging from simple amino acid compositions over physical properties to structure-derived parameters.

In this work, we aimed at selecting predictors that are representative of methodological subgroups described in Table 1 and are implemented in our protein sequence analysis pipeline ANNOTATOR/ANNIE [11-13,27]. These are CAST [23], DisEMBL [22], DISOPRED2 [24], IUPred [25] and SEG [26,42]. These predictors were made available for local installation by the authors.

CAST [23] is a method based on a multi-pass Smith-Waterman comparison of the query sequence against a database of 20 degenerate protein sequences (homopolymers), by using a threshold value associated to a scoring function to identify and mask low complexity regions. Its default threshold has been optimized for BLAST homology searches.

DisEMBL [22] is based on artificial neural networks trained for predicting three different definitions of disorder: coils, hot loops and missing coordinates as in Remark 465. Each predictor has a default value for the minimum score that a residue must have to label the segment as disordered.

DISOPRED2 [24] uses a support vector machine to analyze sequence profiles generated by PSI-BLAST and hence utilizes evolutionary information about the "conservation" of the disorder properties in homologues. The false positive rate threshold can be set at the DISOPRED2 server [43] in discrete intervals from 1% to 10%. Its default value is 5%.

IUPred [25] estimates the total pairwise interaction energy, based on a quadratic form in the amino acid composition of the protein. In this study, we considered IUPred long predictions of any length, once the residue score was above the threshold. IUPred short is suited for predicting short disordered regions, such as missing residues in crystallographic structures.

SEG [26] provides a measure of compositional complexity of a segment of sequence and divides sequences into contrasting segments of low complexity and high complexity. Here, we used the three recommended window sizes (12, 25 and 45) when running SEG. We also applied, as default parameters for the trigger and extension cutoffs, the "medium" mode defined by Sonnhammer and Wootton [42].

A summary on the major differences between the methods benchmarked in this study can be seen in Table 1. As some methods have more than one predictor (DisEMBL, IUPred and SEG), the total number of predictors considered in this study was ten.

### Performance evaluation

All the algorithms benchmarked here were used as a binary classifier for disorder prediction (see Tables 3 to 9 for complete list). In summary, residues were assigned as either disordered or ordered and benchmarked against two main datasets (SL and Remark 465) to compute the four measurements as in the contingency table of Figure 3. In order to test the performance of the algorithms at varying levels of specificity, different thresholds, tuneable according to each method, were used to define disorder and order classification, as described in the section above.

Our approach to measure performance per residue follows the traditional route. Alternatively, one might look after matches between predicted and annotated segments. This path faces several challenges: First, the experimental information does not allow the precise determination of the segment boundaries as reported in the database of disordered regions. Second, the evaluation of matches between predicted and annotated disordered segments requires the introduction of additional parameters such as minimal segment overlap etc. depending on the choice of the evaluators and, therefore, the comparison of methods becomes less objective. And third, some normalization of the length of segments could be required since longer segments are naturally easier to hit by a predictor than shorter ones.

The coefficient, displayed in Figure 3 and known as Matthews Correlation Coefficient (MCC) in the field of secondary structure prediction [34], gives a value between -1 and +1 for the correlation between observation and prediction. In the case of independent variables, a value of 0 is expected for predictions no better than random. A value of -1 indicates total disagreement between observation and prediction, while +1 indicates total agreement. The MCC stores all performance information being easily calculated from the four values (TP, FP, TN and FN), promptly obtained from the contingency table of Figure 3. There are various measurements that can be obtained from combinations of all these four values, but the MCC provides a good performance summary into a single number [44].

The probability excess is an additional measurement that is directly related to the minimal distance from a point in ROC space to the diagonal line corresponding to random predictions (FPR = TPR), that ranges from 0 (random prediction) to 1 (perfect prediction). However, differently from previous work [45], in our SL dataset, the amount of residues annotated as order and disorder is quite comparable (Table 2) and, hence, probability excess as well as MCC can be used.

Receiver operating characteristics (ROC) curves were generated for all algorithms using the two different datasets. These curves are a useful graphing method in evaluating the algorithms' performance [32]. For each algorithm, we evaluated different thresholds and used them as cut-off values for the binary classification. Figure 4 shows the receiver operating characteristic (ROC) curves for ten different classifiers against the two datasets (SL and Remark 465).

The area under the ROC curve (AUC) was also computed using the trapezoid rule [46]. This result can be seen as a Table in Additional file 3.

### Combining the predictors

As the predictors use different methodologies and are trained with diverse datasets, it is not surprising that they produce slightly different outcomes. In this work, we also evaluated the performance of pairs of algorithms. For each pair, we considered consensus and complementary predictions, each under the selection of two sets of parameters. The first set of parameters was chosen so that the individual methods reproduced the same level of specificity at a false positive rate of 0.05, while for the second set the individual methods produced the highest MCC (see Tables 7 and 8). For the consensus prediction, only those residues simultaneously predicted as disordered by both methods were considered as a prediction, while in the complementary case, any prediction was taken into account. In this way, combining ten individual methods, executed under different parameters, resulted in 162 data points in ROC space (Figure 5).

## List of abbreviations used

MCC: Matthews Correlation Coefficient; PE: probability excess; SL: short and long disordered regions; LD40: long disordered regions (length 40 and above); ROC: Receiver operating characteristics

## Competing interests

The authors declare that they have no competing interests.

## Authors' contributions

FLS wrote the manuscript and produced and analyzed the results. SMS participated in the design of the study and analysis of the results. GS, SMS and FE provided feedback throughout the work and revised the manuscript. HSO and TG implemented the benchmark results into the in-house sequence annotation suite (ANNOTATOR/ANNIE). All authors have read and approved the final manuscript.

## Supplementary Material

Additional file 1 **SL dataset**. The SL dataset comprises DisProt r4.5 sequences re-annotated to consider short and long disordered residues, as well as ordered ones. The file is in fasta format, where the amino acid sequence is represented in single letter code and the one line header about the corresponding sequence starts with the symbol ">". The annotation of disordered and ordered regions follows the DisProt description, where the disordered regions are denoted by the symbol "#", while ordered ones are denoted by the symbol "&", followed by the starting and the end residues of the respective region (e.g. #1-10 &11-70 #71-100; where residues from 1 to 10 and 71 to 100 are disordered, while 11-70 are ordered). Click here for file

Additional file 2 **Remark 465 dataset**. The Remark 465 dataset comprises a set of sequences from DisProt r4.5 where at least one structural domain was found in the sequence. Residues annotated under Remark 465 in the PDB were here annotated as disordered. Consequently, the Remark 465 dataset comprises mainly short disordered regions. The file is in fasta format, where the amino acid sequence is represented in single letter code and the one line header about the corresponding sequence starts with the symbol ">". The annotation of disordered and ordered regions follows the DisProt description, where the disordered regions are denoted by the symbol "#", while ordered ones are denoted by the symbol "&", followed by the starting and the end residues of the respective region (e.g. #1-10 &11-70 #71-100; where residues from 1 to 10 and 71 to 100 are disordered, while 11-70 are ordered). Click here for file

Additional file 3 **Supplementary Table and Figures 1 and 2**. Click here for file
