# Supplementary material for: Parameterization of disorder predictors for large-scale applications requiring high specificity by using an extended benchmark dataset
Source: BMC Genomics. 2010 Feb 10;11(Suppl 1):S15. doi: 10.1186/1471-2164-11-S1-S15 (PMC2822529; doi:10.1186/1471-2164-11-S1-S15)
Supplement: Additional file 3 — Supplementary Table and Figures 1 and 2. [file 1471-2164-11-S1-S15-S3.doc]

# Supplementary Material

## Figure 1 - Random predictors

Examples of how the Matthews Correlation Coefficient (MCC) and probability excess (PE) of random predictors follow equal ranking independent of the class ratio (positive:negative), when the specificity level is kept constant (a-c). At different specificity levels (variable FPR), measurements such as MCC and PE don’t correlate with the predictor’s sensitivity (d-f).


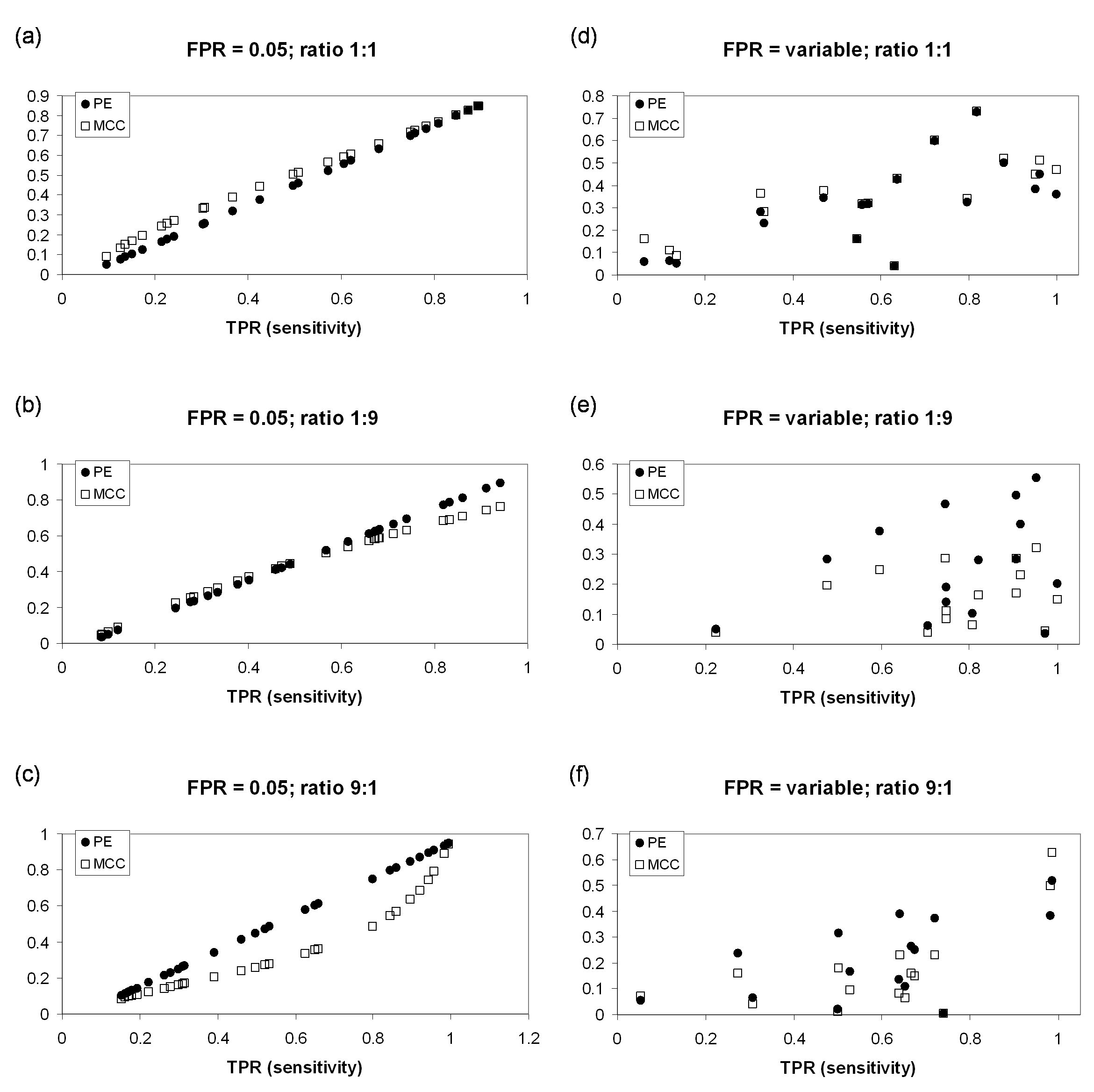


## Figure 2 - IUPforest-L in ROC space

(a) Benchmark against the modified SL dataset (LD40) to have in the positive set the regions of length 40 and above only. (b) Benchmark against Remark 465 dataset. Filled circles are the points where IUPforest-L was executed at the setting stating “*query sequences may contain many long disordered regions*”, while the empty squares are the results under the setting “*query sequences may contain only a few long disordered regions*”. Labels refer to the settings used by the authors that reflect a certain false positive rate benchmarked with their dataset. Dotted lines are the straight continuation of the last measurable data point for DisEMBL predictors to point (1,1) in ROC space.

## Supplementary Table - Area Under the Curve (AUC)

Following the suggestion of one of the reviewers, the area under the curve (AUC) was calculated for each method benchmarked with the three datasets. Note that all curves were connected to the point (1,1) in the graph. In this way, values for curves with dotted lines in Figure 4 of main text could be underestimated.

| Method | SL dataset | Remark 465 | LD40 |
| --- | --- | --- | --- |
| DISOPRED2 | 0.842 | 0.800 | 0.852 |
| IUPred long | 0.841 | 0.719 | 0.869 |
| IUPred short | 0.831 | 0.755 | 0.847 |
| CAST | 0.779 | 0.661 | 0.807 |
| SEG45 | 0.754 | 0.612 | 0.797 |
| SEG25 | 0.732 | 0.634 | 0.761 |
| DisEMBL Coils | 0.699 | 0.658 | 0.709 |
| SEG12 | 0.681 | 0.619 | 0.698 |
| DisEMBL Rem465 | 0.661 | 0.638 | 0.667 |
| DisEMBL Hotloops | 0.656 | 0.627 | 0.656 |
